# Recurrent Stroke Following Left Atrial Appendage Closure in Patients with Breakthrough Stroke: A Systematic Review and Meta-Analysis

**DOI:** 10.3390/jcm15187320

**Published:** 2026-09-21

**Authors:** Tommaso Bini, Marco Gamardella, Antanas Gasys, Roberto Galea, Laurent Roten, David Seiffge, Lorenz Räber, George CM Siontis

**Affiliations:** 1 Department of Cardiology, Inselspital, Bern University Hospital, University of Bern, 3010 Bern, Switzerland; 2 Graduate School for Health Sciences, University of Bern, 3012 Bern, Switzerland; 3 Department of Cardiology, Hospital Centre of Biel, 2502 Biel, Switzerland; 4 Department of Neurology, Inselspital, Bern University Hospital, University of Bern, 3010 Bern, Switzerland; 5 Institute of Social and Preventive Medicine (ISPM), University of Bern, 3012 Bern, Switzerland

**Keywords:** breakthrough stroke, cerebrovascular accident, left atrial appendage occlusion, LAAO, anticoagulation failure

## Abstract

**Objective**: To determine the incidence of recurrent ischemic stroke following percutaneous left atrial appendage closure in patients with previous thromboembolic events despite oral anticoagulation. **Methods**: We conducted a systematic review and meta-analysis of studies of any design reporting ischemic stroke recurrence after left atrial appendage closure in patients with prior ischemic stroke despite oral anticoagulation therapy. The literature search was conducted in MEDLINE, Embase, and the Cochrane Library for studies published between 1 January 2010 and 31 December 2025. Data on study design, population characteristics, indexed intervention, and outcomes were extracted from each included report. The primary outcome was ischemic stroke occurring after left atrial appendage closure, as defined by each individual study. A pooled incidence rate was estimated using a random-effects model to account for anticipated between-study heterogeneity. The study is registered in Open Science Framework. The Risk of Bias in Non-randomized Studies of Interventions assessment tool was used to assess the risk of bias. **Results**: Overall, eight studies of a total of 1091 patients met the eligibility criteria. Patients were elderly (mean age range 71.8 to 78.1 years), 33% to 53% were women, and thromboembolic risk was consistently high (mean CHA_2_DS_2_-VASc scores ≥ 5.0). The risk of bias across the included studies was either moderate or serious. Antithrombotic therapy after left atrial appendage closure varied considerably across the included studies. In one study, all patients were discharged on dual antiplatelet therapy, whereas in five studies >70% received oral anticoagulation alone or in combination with antiplatelet therapy at discharge. In six studies, antithrombotic therapy at follow-up was reported, with the proportion of patients on oral anticoagulation alone or in combination with antiplatelet therapy at follow-up ranging from 8.1% to 100% in individual studies. Follow-up duration ranged from 1.0 to 3.1 years. At a mean follow-up of 17.7 months the pooled incidence of the ischemic stroke recurrence rate following left atrial appendage closure was 3.1% per year (95% confidence interval 2.35–4.20%). **Conclusions**: Among elderly patients with prior ischemic stroke despite oral anticoagulation, the recurrence rate of ischemic stroke following left atrial appendage closure was 3.1% per year. Given the residual risk of stroke recurrence and the substantial heterogeneity in post-procedural antithrombotic management observed across studies, prospective randomized trials are urgently needed to define both the protective impact of left atrial appendage closure and the optimal antithrombotic strategy in this high-risk population.

## 1. Introduction

Atrial fibrillation (AF) is the most common cardiac arrhythmia globally [1]. The presence of AF places patients at a fivefold increased risk of thromboembolic events, particularly ischemic stroke. Accordingly, in patients with AF who are at elevated thromboembolic risk, oral anticoagulation (OAC) carries a Class of Recommendation I with a Level of Evidence A recommendation from both European and American cardiology societies [2,3].

Although OAC normally represents an effective treatment for preventing thromboembolic events, a small percentage of patients with AF (1–2% per year) may experience recurrent cardioembolic stroke despite appropriate OAC therapy, a phenomenon referred to as “breakthrough stroke” [4,5,6,7,8]. In this population, switching anticoagulants and/or adding antiplatelet therapy has not been shown to significantly reduce the risk of recurrent thromboembolic events and is not recommended by European guidelines (Class III recommendation) [9,10,11,12]. In addition, evidence from prospective observational studies and post-hoc analyses of the pivotal randomized controlled trials (RCTs) comparing direct oral anticoagulants (DOACs) with vitamin K antagonists (VKAs) in patients with atrial fibrillation (AF) indicates that patients who experience a breakthrough stroke remain at a very high-risk of recurrent ischemic stroke with a frequency between 5–9% in the first year [13,14,15,16]. These findings identify this subgroup as a particularly vulnerable population with an unmet clinical need. Therefore, novel treatment strategies are required to reduce the risk of recurrent stroke and improve long-term outcomes in these high-risk patients.

The LAAOS III trial, in which patients with AF undergoing cardiac surgery were randomized to concomitant surgical left atrial appendage closure (LAAC) in addition to continuation of OAC therapy, demonstrated a reduced risk of thromboembolic events in patients randomized to LAAC. Similarly, percutaneous LAAC has emerged in recent years as a potential therapeutic add-on to OAC therapy in patients experiencing breakthrough stroke [17]. Although the underlying mechanisms of ischemic stroke in patients with AF receiving OAC remain incompletely understood and are likely multifactorial, several observational studies have reported promising outcomes, particularly when LAAC was performed alongside continued OAC [18,19,20,21]. To our knowledge, no study has systematically evaluated the impact of LAAC on recurrent ischemic stroke in this specific patient population.

In light of the limited available evidence on this topic, we conducted a systematic review and meta-analysis to determine the incidence of recurrent ischemic stroke following LAAC in patients who previously experienced ischemic stroke despite ongoing OAC therapy.

## 2. Methods

The protocol for the systematic review was prespecified and published on Open Science Framework (OSF) (https://osf.io/jq6wx, accessed on 13 September 2026). The Preferred Reporting Items for Systematic reviews and Meta-Analysis Protocols (PRISMA) reporting guidelines were followed (Supplementary PRISMA 2020 CHECKLIST) [22]. The review was not prospectively registered in a systematic review registry.

### 2.1. Search Strategy

We performed a systematic computerized literature search covering the period from 1 January 2010 (corresponding to the period following the introduction of LAAC as a potentially viable alternative to OAC for stroke prevention) to 31 December 2025 in MEDLINE, Embase, and Cochrane Central Register of Controlled Trials (CENTRAL) by using a combination of medical subject headings and free text (Supplementary Materials). In addition, we manually searched for eligible studies in the reference lists of the potentially eligible publications, and relevant meta-analyses in the field that had been previously identified through the above search. Two reviewers (TB and MG) independently screened abstracts and performed full-text reviews. Any disagreement was resolved through discussion or by consulting a third reviewer.

### 2.2. Eligibility Criteria

We applied very broad eligibility criteria. Studies were eligible for inclusion if they met the following two criteria: (1) reported the results of a study of any original design, and (2) reported the incidence of the outcome of stroke following LAAC in patients with a previous thromboembolic event under oral anticoagulation. No restrictions were considered in terms of publication year, language or sample size of the study. Case reports and case series were excluded. When potentially overlapping study populations were identified in different publications (according to the period of recruitment and involved institutions), we gave preference to the most recent publication or the report providing data on antithrombotic treatment at discharge and throughout the follow-up.

### 2.3. Data Extraction

Two reviewers (TB and MG) independently extracted data using a standardized, piloted data extraction form. Disagreements were resolved by consensus or by consultation with a third reviewer (RG). Study characteristics and information on the study population, LAAC procedure and clinical outcomes were extracted and summarized on study level. For each eligible study, we recorded study-related information (first author, year of publication, recruiting center, recruitment period, study period, study design, sample size, funding source and follow-up time) and summary descriptive of patients’ characteristics and LAAC-related information. Data on post-procedural antithrombotic management were also extracted, encompassing the use of OAC alone or in combination with antiplatelet therapy (APT) at discharge and at follow-up. Where available, patients receiving a DOAC and those receiving a VKA were recorded separately to allow for subgroup characterization.

The primary outcome of interest was ischemic stroke occurring after LAAC, as defined by each individual study. For each included report, the number of outcome events and the corresponding follow-up time were extracted to allow calculation of incidence rates. We also extracted descriptive information on other clinical outcomes, including all-cause death, cardiovascular death, hemorrhagic stroke, and periprocedural safety.

### 2.4. Risk of Bias Assessment

Two investigators (TB and MG) independently reviewed the studies and judged the risk of bias on study level. To assess the risk of bias in non-randomized studies, we used the Risk of Bias in Non-randomized Studies of Interventions (ROBINS-I) assessment tool [23].

### 2.5. Statistical Analysis

We summarized descriptive information of the included studies. For each study, the annualized incidence rate of ischemic stroke recurrence was calculated as the number of events divided by the total patient follow-up time expressed in person-years. When annualized incidence rates were not directly reported, they were derived by dividing the number of recurrent ischemic stroke events by an approximated total person-time at risk, calculated as the number of participants and the reported mean or median follow-up duration. The pooled incidence rate of any stroke occurring after LAAC, with corresponding 95% confidence interval (CI), was estimated using a random-effects model with the Hartung–Knapp–Sidik–Jonkman (HKSJ) adjustment, which was applied to produce more conservative confidence intervals under conditions of high between-study heterogeneity. A forest plot was used to visually present the study-specific and pooled incidence rates with corresponding 95% CIs. Heterogeneity was quantified using the I^2^ statistic (values of 25%, 50%, and 75% representing low, moderate, and high heterogeneity, respectively), and τ^2^, representing the estimated between-study variance [24,25,26]. All analyses were performed using R version 4.5.2 (R Foundation for Statistical Computing, Vienna, Austria).

## 3. Results

Overall, 502 records were scrutinized for eligibility. Three hundred ninety-nine were assessed on title and abstract level; 43 were evaluated in full text. Seven studies were excluded for the presence of overlapping populations [18,19,20,21,27,28,29]. Finally, we identified 8 studies (1091 patients) that fulfilled the prespecified inclusion criteria [30,31,32,33,34,35,36,37]. The study characteristics of the included studies are reported in Table 1 and Table 2. Five studies were performed in Europe (n = 283), one in Asia (n = 346), one in North America (n = 29), and one in more continents (n = 433). All included studies were published in peer-reviewed journals between 2019 and 2025. Among the eight included studies, prospective and retrospective designs were equally represented (n = 4 each, 50%), and most were conducted across multiple centers (n = 5, 63%).

The detailed risk of bias assessment is available in the online supplementary material (Table S1). Overall, risk of bias was difficult to assess mainly due to the absence of detailed reporting and the methodological diversity of the included studies. No study was judged to be at critical risk of bias. The studies were equally judged (50%) at serious and at moderate risk of bias. Within each domain of interpretation of the ROBINS-I tool, all the non-randomized studies were at low risk of bias with regard to the domains of bias in classification of intervention and bias due to deviations from intended intervention.

The mean age ranged from 71.8 to 78.1 years, with 33% to 53% of participants being women. Thromboembolic risk was consistently high, with mean/median CHA_2_DS_2_-VASc scores ranging from 5.0 to 6.0. Specifics about the recruitment period and funding sources of each study are reported in the online supplementary material (Table S2). In three studies the baseline as well as follow-up breakthrough strokes were assessed by a neurologist, after excluding other etiologies [30,34,36]. Only one study reported the time between index stroke and LAAC, with a median interval of approximately 4 months [36]. The presence of LAA thrombus at the time of the procedure was reported in 2 studies occurring in 12.8% and 18% of patients, respectively [33,35].

**Figure 1:**
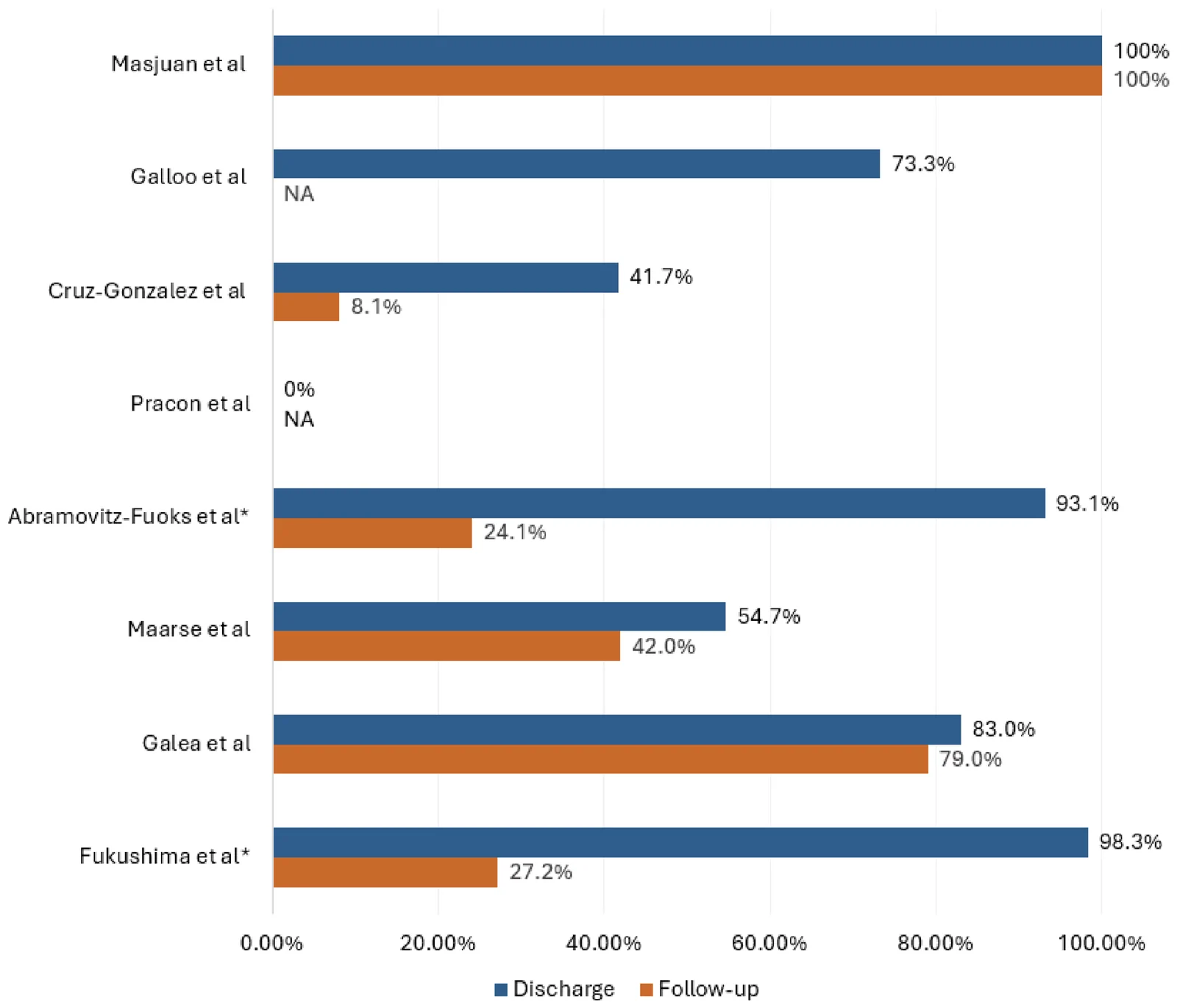
Oral anticoagulation (OAC) alone or in combination with antiplatelet therapy (APT) at discharge and at follow-up. Percentage of patients on OAC alone or in combination with APT at discharge and at the latest follow-up. Studies are sorted by year of publication. * patients on OAC alone or in combination with APT at 1 year were reported. NA: not available. Studies are sorted by year of publication [30,31,32,33,34,35,36,37].

The antithrombotic treatment at discharge was reported in all studies. The proportion of patients discharged with OAC alone or in combination with APT was highly variable among the studies, ranging from 0% to 100%. In one study all patients were discharged on dual antiplatelet therapy (DAPT) [33]. In five studies patients discharged on OAC alone or in combination with APT were >70% of the individual cohort and in three of them >90% [30,31,34,36,37]. OAC use at follow-up was reported in six studies [30,32,34,35,36,37]. The proportions of patients receiving OAC alone or in combination with APT at discharge and at follow-up in the individual studies are presented in Figure 1. In four studies, the type of OAC at discharge was specified, and in two of these studies the distribution at follow-up was available [30,31,32,35].

Periprocedural safety was reported in 7 studies and ranged from 0% to 7.8% [30,31,32,34,35,36,37]. In the remaining study, periprocedural safety was not specifically reported; however, procedure-related clinically significant bleeding occurred in 5.1% of patients [33].

All-cause mortality was reported in all included studies, ranging from 0% to 20%; in seven of eight studies, the rate was ≤7.7% (Table S6). Cardiovascular death was reported in three studies: two recorded no cardiovascular deaths, while one reported a rate of 3.5%; the remaining studies did not report this outcome [30,31,37]. Hemorrhagic stroke during follow-up was reported only by 2 studies, with rates of 0% and 0.3%, respectively [31,37].

**Figure 2:**
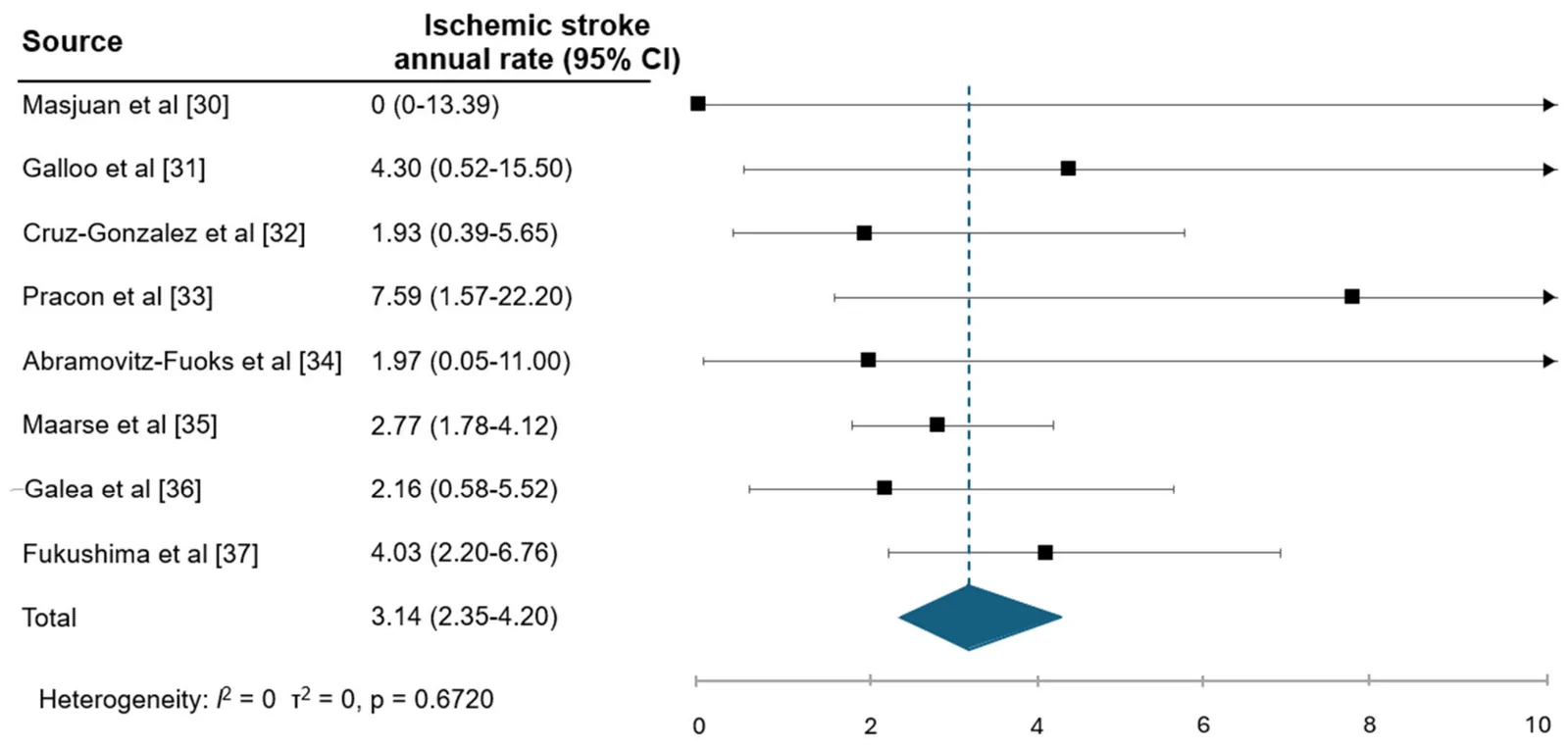
Ischemic stroke recurrence rate per year. Forest plot for ischemic stroke recurrence rate using a random-effects model meta-analysis with the Hartung–Knapp–Sidik–Jonkman (HKSJ) adjustment. Studies are sorted by year of publication [30,31,32,33,34,35,36,37].

The ischemic stroke recurrence rate after LAAC in patients with prior ischemic stroke despite OAC therapy was reported in all but two studies. In one of these, it was presented as the incidence per 100 patient-years, and in the other as the annualized event rate [34,35]. After converting all reported rates to annualized values, the incidence of ischemic stroke recurrence across individual studies ranged from 1.9% to 7.6% per year (Table 2). The pooled annualized incidence rate was 3.14% per year (95% CI 2.3–4.2%), with no statistically detected heterogeneity between studies (I^2^ = 0.0%; τ^2^ = 0.0) (Figure 2). The limited number of studies and the form of available evidence did not allow us to further investigate possible causes of heterogeneity.

## 4. Discussion

This systematic review and meta-analysis represents, to our knowledge, the most comprehensive synthesis of evidence on ischemic stroke recurrence following LAAC in patients with prior ischemic stroke despite OAC therapy. Across eight studies encompassing 1091 patients, we observed a pooled annualized ischemic stroke recurrence rate of 3.14% per year (95% CI 2.3–4.2%). The included population was consistently elderly and at high thromboembolic risk, with mean CHA_2_DS_2_-VASc scores ≥ 5.0 across all studies, underscoring the clinical complexity of this patient group. The substantial variability in post-procedural antithrombotic management, ranging from DAPT in one study to predominantly OAC-based regimens in others, with follow-up OAC use spanning from 8.1% to 100%, makes it difficult to attribute the observed recurrence rates to LAAC itself rather than to concomitant antithrombotic therapy.

Stroke represents one of the leading causes of disability and mortality in patients with AF [38]. Consequently, effective stroke prevention in this population is of paramount importance. Although OAC is an established and highly effective therapy for stroke prevention in AF, a subset of patients may nevertheless experience OAC failure. Several studies have documented a substantial rate of ischemic stroke recurrence among patients who suffer a stroke despite OAC therapy [14,15,16,39,40,41]. In this context, one study reported an annualized recurrence rate of 8.9% among 1195 patients with a median CHA_2_DS_2_-VASc score of 5, underscoring not only the elevated risk of recurrence in this population but also the potential underestimation of stroke risk when relying solely on the CHA_2_DS_2_-VASc score [13]. A CHA_2_DS_2_-VASc score of 5 in an OAC-naïve patient with AF would typically correspond to an annual stroke risk of approximately 7.2%. However, when considering the recurrence rates observed in patients who experienced stroke despite OAC, it becomes evident that the CHA_2_DS_2_-VASc score may not adequately capture thromboembolic risk in this particularly high-risk subgroup [42,43,44]. Furthermore, a recent meta-analysis including 10,989 patients across six studies—one of which comprised pooled individual participant data from five pivotal RCTs—reported a pooled incidence rate of recurrent ischemic stroke of 7.2% per year among patients with a prior stroke despite OAC [45].

When compared with the previously reported incidence rate of 7.2% per year, our annualized rate of 3.14% appears numerically lower. This comparison is indirect and hypothesis-generating only: it derives from different cohorts and cannot be interpreted as evidence that LAAC reduced recurrent stroke compared with continued OAC alone, particularly given the relatively high risk of bias across the included studies. In addition, several important aspects remain unresolved and warrant further investigation. First, the optimal antithrombotic regimen following percutaneous LAAC has yet to be clearly established, particularly in patients with ischemic stroke. In the study conducted by Pracon et al., all patients were discharged on DAPT, which was associated with an annualized recurrence rate of 7.59% [33]. In contrast, in the studies by Masjuan et al., Abramovitz-Fuoks et al., and Fukushima et al., in which almost all patients were continued on OAC therapy, the reported annualized recurrence rates were 0%, 1.97%, and 4.03%, respectively. These findings, backed by the results of the LAAOS III trial, suggest that a combined strategy consisting of percutaneous LAAC together with continued OAC may represent a more effective therapeutic approach in this high-risk population [30,34,37]. The high degree of heterogeneity in post-procedural antithrombotic therapy does not allow our findings to be interpreted with certainty. Nevertheless, any assumptions regarding the optimal post-procedural antithrombotic regimen remain purely speculative at this stage and warrant confirmation in RCTs. Second, the question of whether OAC can or should be discontinued and if yes with or without additional single antiplatelet therapy remains an important and unresolved issue. Across the included studies, discontinuation rates varied considerably. In the study by Abramovitz-Fuoks et al., antithrombotic therapy was de-escalated to single antiplatelet therapy (SAPT) at one year in 62.2% of patients. Similarly, Fukushima et al. discontinued OAC in 72.8% of patients at one year [34,37]. However, in the LAAOS III trial, surgical LAAC performed in addition to ongoing OAC provided incremental stroke prevention benefits compared with OAC alone [17]. Consistently, in the study by Masjuan et al., all patients remained on OAC throughout follow-up, with no ischemic recurrences observed during a mean follow-up of 17.4 months [30].

To place our findings in context, the annual stroke risk in atrial fibrillation patients with CHA_2_DS_2_-VASc scores ≥ 5 on OAC therapy has been reported to range from approximately 3% to 6% per year, suggesting that LAAC may offer a comparable degree of stroke prevention in patients who have already failed OAC. However, these findings highlight both the potential and the current limitations of the available evidence base in this challenging population. A strategy combining percutaneous LAAC with indefinite continuation of OAC could hypothetically offer additional protection against recurrent thromboembolic events in this high-risk population. Nevertheless, this hypothesis requires confirmation in dedicated and adequately powered randomized controlled trials. The ELAPSE (NCT05976685) and ADD-LAAO (NCT06869811) trials are investigator-initiated, international, superiority RCTs comparing continued OAC alone with the combination of LAAC plus ongoing OAC [46]. Both studies aim to clarify whether adding percutaneous LAAC to sustained anticoagulation confers incremental benefit in patients with breakthrough stroke. Similarly, the LAAOS-4 trial (NCT05963698) is a sponsored, international, superiority RCT comparing OAC alone with LAAC plus continued OAC in patients with AF at very high thromboembolic risk (CHA_2_DS_2_-VASc ≥ 4), although not necessarily with a prior breakthrough stroke. Collectively, these trials are expected to provide crucial evidence to inform the optimal management strategy for this challenging and high-risk population.

## 5. Limitations

Several limitations must be acknowledged when interpreting the findings of the present study. First, the absence of a control group substantially limits the strength of the conclusions that can be drawn and precludes direct comparisons with alternative therapeutic strategies. Second, only a rather small number of studies fulfilled the predefined inclusion criteria, which may restrict the generalizability of our findings to broader clinical populations. Third, in only a minority of the included studies were stroke events adjudicated by a neurologist. This may have introduced selection and reporting bias, potentially affecting the accuracy and consistency of outcome assessment. Fourth, all studies meeting the inclusion criteria were observational in nature. The inherent limitations of observational designs, including residual confounding and selection bias, must therefore be considered when interpreting the results. Fifth, although we did not detect statistically significant heterogeneity, the relatively small number of included studies reduces the statistical power to identify moderate between-study heterogeneity. Therefore, meaningful variability across studies cannot be excluded, particularly in light of the wide confidence intervals observed. Finally, post-procedural antithrombotic management following percutaneous LAAC varied considerably across studies and, in some cases, was not reported. This heterogeneity precluded the performance of sensitivity analyses stratified according to antithrombotic therapy and limited our ability to determine the relative contribution of different pharmacological strategies to the observed outcomes.

## 6. Conclusions

Among elderly, high-risk patients with prior ischemic stroke despite OAC, the recurrence rate of ischemic stroke following LAAC was 3.14% per year. Given the residual risk of stroke recurrence and the substantial heterogeneity in post-procedural antithrombotic management observed across studies, prospective randomized controlled trials are urgently needed to define both the protective impact of LAAC and the optimal antithrombotic strategy in this high-risk population.

## Figures and Tables

**Table 1:** Characteristics of the included studies.

Source	Year of Publication	Location	Study Design	Sample Size	Follow-Up, Months (Mean ± SD)	OAC at Discharge, %
Masjuan et al. [30]	2019	Spain	PS-SC	19	17.4 ± 11.5	100.0
Galloo et al. [31]	2020	Belgium	RS-MC	15	37.2 ± 32.4	73.3
Cruz-Gonzalez et al. [32]	2020	Spain	RS-MC	115	16.2 ± 12.2	41.7
Pracon et al. [33]	2022	Poland	PS-SC	39	12.2 (11.8–12.9) ^1^	0.0
Abramovitz-Fuoks et al. [34]	2024	USA	RS-SC	29	21.0 ± 12.0	93.1
Maarse et al. [35]	2024	International	PS-MC	433	24.0	54.7
Galea et al. [36]	2025	International	RS-MC	95	23.4 (12.3–24.0) ^1^	83.0
Fukushima et al. [37]	2025	Japan	PS-MC	346	12.1 (8.0–13.8) ^1^	98.3

^1^ Values are expressed as median IQR. OAC: Oral Anticoagulation; PS: Prospective; RS: Retrospective; SC: Single-center; MC: Multicenter.

**Table 2:** Baseline characteristics and stroke rate at follow-up in case and control groups at study-level.

Source	Age (Mean ± SD)		Female Gender, %		CHA_2_DS_2_-VASC (Mean ± SD)		HAS-BLED (Mean ± SD)		Hypertension, %		Diabetes Mellitus, %		Stroke Rate ^1^, %	
	Case	Control	Case	Control	Case	Control	Case	Control	Case	Control	Case	Control	Case	Control
Masjuan et al. [30]	72.1 ± 9.6	/	37.0	/	5.3 ± 1.5	/	1.73 ± 1.2	/	73.6	/	21.0	/	0.00	/
Galloo et al. [31]	78.1 ± 5.8	/	53.3	/	6.0 ± 1.2	/	5.0 ± 1.2	/	80.0	/	20.0	/	4.30	/
Cruz-Gonzalez et al. [32]	73.8 ± 10.2	75.1 ± 8.2	34.8	38.5%	5.5 ± 1.5	4.3 ± 1.6	3.9 ± 1.3	3.1 ± 1.2	80.9	83.2	29.6	29.2	1.93	0.47
Pracon et al. [33]	73.0 (62.0–77.0) ^2^	74.0 (68.0–81.0) ^2^	48.7	41.7	5.0 (3.0–6.0) ^2^	4.0 (3.0–5.0) ^2^	2.0 (1.0–3.0) ^2^	3.0 (2.0–3.0) ^2^	92.3	83.3	30.8	30.1	7.59	0.63
Abramovitz-Fuoks et al. [34]	73.4 ± 8.7	/	44.8	/	5.9 ± 1.3	/	4.2 ± 0.9	/	96.9	/	51.7	/	1.97	/
Maarse et al. [35]	71.8 ± 9.4	72.7 ± 9.0	39.0	36.0	5.0 ± 1.6	5.1 ± 1.5	2.8 ± 1.3	NA	76.0	82.0	25.0	23.0	2.77	8.89
Galea et al. [36]	74.1 ± 11.4	/	38.0	/	5.4 ± 1.3	/	3.0 ± 1.0	/	77.0	/	28.0	/	2.16	/
Fukushima et al. [37]	76.4 ± 7.9	77.5 ± 7.5	33.2	32.6	6.0 (5.0–7.0) ^2^	4.0 (3.0–5.0) ^2^	3.0 (3.0–4.0) ^2^	3.0 (2.0–3.0) ^2^	82.3	82.0	34.4	37.5	4.03	1.63

^1^ Annual ischemic stroke recurrence rate at follow-up. ^2^ Median IQR.

## Data Availability

The original contributions presented in this study are included in the article/Supplementary Material. Further inquiries can be directed to the corresponding author.

## Supplementary Materials

The supporting information can be downloaded at: https://www.mdpi.com/article/10.3390/jcm15187320/s1, Figure S1: Study Flow Chart; Table S1. Risk of Bias for Non-randomized Studies for Interventions (ROBINS-I); Table S2. Recruitment period and funding; Table S3. Control group population; Table S4. LAAC-Device used; Table S5. Percentage of patients on direct oral anticoagulation (DOAC) or vitamin K antagonist (VKA) at discharge and at follow-up; Table S6. Clinical outcomes at mean/median follow-up; Table S7. Periprocedural safety; Table S8. PRISMA 2020 CHECKLIST.

## Abbreviations

The following abbreviations are used in this manuscript:

AF	Atrial Fibrillation
APT	Antiplatelet Therapy
CENTRAL	Cochrane Central Register of Controlled Trials
CI	Confidence Interval
DAPT	Dual Antiplatelet Therapy
DOAC	Direct Oral Anticoagulant
HKSJ	Hartung–Knapp–Sidik–Jonkman
LAAC	Left Atrial Appendage Closure
OAC	Oral Anticoagulation
PRISMA	Preferred Reporting Items for Systematic reviews and Meta-Analysis Protocols
RCT	Randomized Controlled Trial
ROBINS-I	Risk of Bias in Non-randomized Studies of Interventions
SAPT	Single Antiplatelet Therapy
VKA	Vitamin-K Antagonist
