# Supplementary material for: Recurrent Stroke Following Left Atrial Appendage Closure in Patients with Breakthrough Stroke: A Systematic Review and Meta-Analysis
Source: J Clin Med. 2026 Sep 21;15(18):7320. doi: 10.3390/jcm15187320 (PMC13607750; doi:10.3390/jcm15187320)
Supplement: Supplementary file 1 [file jcm-15-07320-s001.zip › jcm-4538250-supplementary.pdf]

SUPPLEMENTARY MATERIAL

Incidence of recurrent stroke following left atrial appendage closure in patients with ischemic stroke on oral anticoagulation: a systematic review and meta-analysis.

Bini et al

Table of Contents

Study flow-chart	page 1
Search stage	page 2
De-duplication	page 5
Screening stage	page 6
Table S1	page 7
Table S2	page 8
Table S3	page 9
Table S4	page 10
Table S5	page 11
Table S6	Page 12
Table S7	Page 13
Protocol Amendment	Page 14
PRISMA 2020 for abstracts checklist	Page 15
PRISMA 2020 checklist	Page 16

STUDY FLOW-CHART

Study identification and selection.

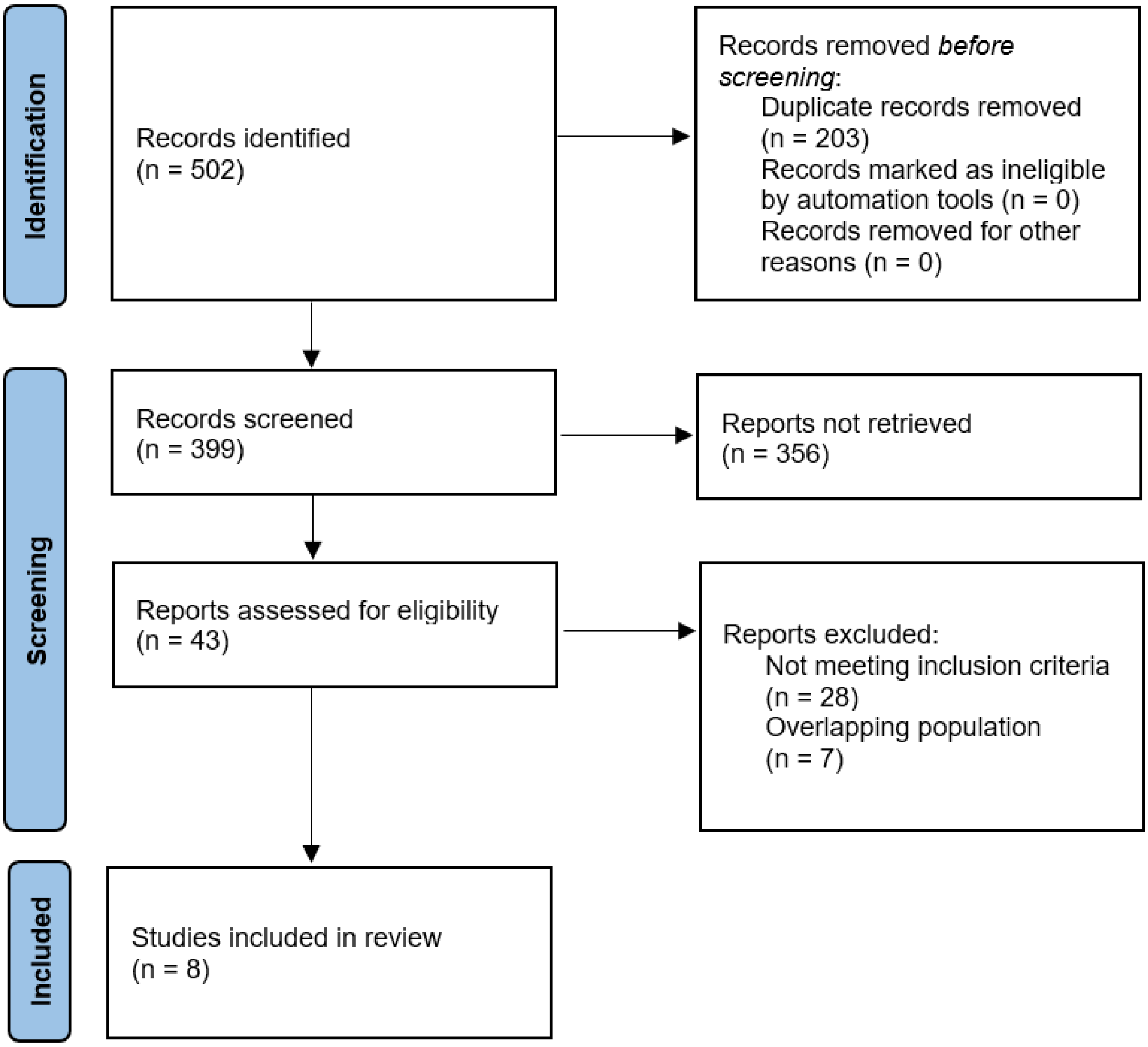

SEARCH STAGE

Database and number of results

**Database/Interface**	**Dates covered**	**Number of records**
Ovid MEDLINE ALL	2010-2026	275
Embase	2010-2026	135
Cochrane Library	2010-2026	92
Total number of records retrieved: **502**		
Total number of records after de-duplication: **399**		

Details of individual research

1st Search Strategy (applied on 07/01/2026)

Ovid MEDLINE® ALL

1. Exp Left Atrial Appendage closure/ or left atrial appendage occlusion/
2. (left atrial appendage adj4 (occlu* or closure)).tw
3. (LAAC or LAAO).tw
4. 2 and (stroke adj4 (recurren* or resistant or thromboembolic or cardioembolic or breakthrough)).mp
5. 3 and (stroke adj4 (recurren* or resistant or thromboembolic or cardioembolic or breakthrough)).mp
6. 2 and (stroke despite anticoagulation).mp
7. 3 and (stroke despite anticoagulation).mp
8. 2 and (anticoagulation failure).mp
9. 3 and (anticoagulation failure).mp
10. or 4-9
11. Limit 10 to year=”2010-Current”

Embase

1. “Left Atrial Appendage closure”/ or “left atrial appendage occlusion”/
2. (percutaneous adj4 (occlu* or closure)).mp.
3. (left atrial appendage or LAAC or LAAO).mp.
4. 2 and 3
5. 4 and (stroke adj4 (recurren* or resistant or thromboembolic or cardioembolic or breakthrough)).mp.
6. 4 and stroke despite anticoagulation.mp.
7. 4 and anticoagulation failure.mp.
8. 5 or 6 or 7
9. Limit 8 to conference abstract status
10. 8 not 9
11. Limit 10 to yr=”2010-Current”

Cochrane Library (CENTRAL)

1. MeSH descriptor: [Left Atrial Appendage Closure] explode all trees
2. “left atrial appendage (closure or occlusion)”:ti,ab,kw
3. “LAAC or LAAO”:ti,ab,kw
4. 2 or 3
5. 4 and (stroke (recurrent or resistant or thromboembolic or cardioembolic or breakthrough)):ti,ab,kw
6. 4 and (stroke despite anticoagulation):ti,ab,kw
7. 4 and (anticoagulation failure):ti,ab,kw
8. 5 or 6 or 7 with Publication Year from 2010 to 2025, in Trials

Correction of the Search Strategy

The search strategy was refined to align with the updated inclusion criteria and to ensure retrieval of key studies known a priori that were not identified using the initial search strategy.

2nd Search Strategy (applied on 12/01/2026)

Ovid MEDLINE® ALL

1. Exp Left Atrial Appendage closure/ or left atrial appendage occlusion/ **153**
2. (left atrial appendage adj4 (occlu* or closure)).tw **3796**
3. (LAAC or LAAO).tw **1762**
4. 2 and (stroke adj4 (recurren* or resistant or thromboembolic or cardioembolic or breakthrough)).mp **218**
5. 3 and (stroke adj4 (recurren* or resistant or thromboembolic or cardioembolic or breakthrough)).mp **97**
6. 2 and (despite adj4 anticoagula* or despite OAC).mp **82**
7. 3 and (despite adj4 anticoagula* or despite OAC).mp **51**
8. 2 and (anticoagulation failure or failure of anticoagulation).mp **10**
9. 3 and (anticoagulation failure or failure of anticoagulation).mp **4**
10. or 4-9 **288**
11. Limit 10 to year=”2010-Current” **275**

Embase

1. “Left Atrial Appendage closure”/ or “left atrial appendage occlusion”/ **3670**
2. (percutaneous adj4 (occlu* or closure)).mp. **12068**
3. (left atrial appendage or LAAC or LAAO).mp. **12627**
4. 2 and 3 **1906**
5. 4 and (stroke adj4 (recurren* or resistant or thromboembolic or cardioembolic or breakthrough)).mp. **203**
6. 4 and ((despite adj4 anticoagula*) or (despite OAC)).mp. **55**
7. 4 and (anticoagulation failure or failure of anticoagulation).mp. **6**
8. 5 or 6 or 7 **244**
9. Limit 8 to conference abstract status **98**
10. 8 not 9 **146**
11. Limit 10 to yr=”2010-Current” **135**

Cochrane Library (CENTRAL)

1. MeSH descriptor: [Left Atrial Appendage Closure] explode all trees **9**
2. “left atrial appendage (closure or occlusion)”:ti,ab,kw **414**
3. “LAAC or LAAO”:ti,ab,kw **259**
4. 2 or 3 **443**
5. 4 and (stroke (recurrent or resistant or thromboembolic or cardioembolic or breakthrough)):ti,ab,kw **76**
6. 4 and ((despite NEAR/4 anticoagula*) or (despite OAC)):ti,ab,kw **9**
7. 4 and ((anticoagulation failure) or (failure of anticoagulation)):ti,ab,kw **24**
8. 5 or 6 or 7 with Publication Year from 2010 to 2026, in Trials **92**

DE-DUPLICATION

De-Duplication strategy on EndNote

	Matching Strategy	Duplicates	No. Retrieved
1st Round	Author, Year, Title and Reference Type	**138**	**432**
2nd Round	Author and Title	**2**	**431**
3rd Round	Title	**47**	**407**
4th Round	Author	**16**	**399**
Number of Duplicates: **203**			

SCREENING STAGE

After independent review among two reviewers.

Concluded on 17/01/2026

1st Phase – Screening Titles and Abstracts

Number of studies excluded: **356**

Number retrieved: **43**

2nd Phase – Screening Full texts

Number of studies excluded: **28**

Number retrieved: **15**

- Aarnink et al https://dx.doi.org/10.1016/j.jcin.2024.04.012
- Abramovitz Fuoks et al https://dx.doi.org/10.1136/svn-2024-003143
- Chatani et al https ://dx.doi.org/10.1253/circj.CJ-25-0678
- Chatani et al https ://dx.doi.org/10.1016/j.hrthm.2024.10.061
- Cruz-Gonzalez et al https ://dx.doi.org/10.1016/j.rec.2019.02.013
- Falasconi et al https://dx.doi.org/10.23736/S0031-0808.21.04446-3
- Freixa et al Journal of Invasive Cardiology 2019 Vol. 31 Issue 8 Pages 212-216
- Fukushima et al https://dx.doi.org/10.1016/j.jacep.2025.07.021
- Galea et al https://dx.doi.org/10.1111/ene.70365
- Galloo et al https://dx.doi.org/10.1080/17843286.2020.1821494
- Maarse et al https ://dx.doi.org/10.1001/jamaneurol.2024.2882
- Margonato et al https://dx.doi.org/10.1111/ene.13894
- Masjuan et al https://dx.doi.org/10.1111/ene.13894
- Pracon et al https ://dx.doi.org/10.1016/j.ijcard.2022.02.030
- Preda et al https://dx.doi.org/10.1016/j.ijcard.2024.132875

3rd Phase – Screening Overlapping population

Number of studies excluded: **2**

Number retrieved: **8**

- Abramovitz Fuoks et al https://dx.doi.org/10.1136/svn-2024-003143
- Cruz-Gonzalez et al https ://dx.doi.org/10.1016/j.rec.2019.02.013
- Fukushima et al https://dx.doi.org/10.1016/j.jacep.2025.07.021
- Galea et al https://dx.doi.org/10.1111/ene.70365
- Galloo et al https://dx.doi.org/10.1080/17843286.2020.1821494
- Maarse et al https ://dx.doi.org/10.1001/jamaneurol.2024.2882
- Masjuan et al https://dx.doi.org/10.1111/ene.13894
- Pracon et al https ://dx.doi.org/10.1016/j.ijcard.2022.02.030

Table S1. Risk of Bias for Non-randomized Studies for Interventions (ROBINS-I)

	Risk of bias due to confounding	Risk of bias in classification of interventions	Risk of bias in selection of participants into the study (or into the analysis)	Risk of bias due to deviations from intended interventions	Risk of bias due to missing data	Risk of bias arising from measurement of the outcome	Risk of bias in selection of the reported result	Overall judgment
**Masjuan et al**								
**Galloo et al**								
**Cruz-Gonzalez et al**								
**Pracon et al**								
**Abramovitz Fuoks et al**								
**Maarse et al**								
**Galea et al**								
**Fukushima et al**								

Judgement	Interpretation
**Low risk of bias**	There is little or no concern about bias with regard to this domain.
**Moderate risk of bias**	There is some concern about bias with regard to this domain, although it is not clear that there is an important risk of bias.
**Serious risk of bias**	The study has some important problems in this domain: characteristics of the study give rise to a serious risk of bias.
**Critical risk of bias**	The study is very problematic in this domain: characteristics of the study give rise to a critical risk of bias, such that and the result should generally be excluded from evidence syntheses.

Table S2. Recruitment period and funding

Source	Recruitment period	Funding
Masjuan et al	01/2015 – 07/2018	None
Galloo et al	06/2009 – 10/2019	None
Cruz-Gonzalez et al	12/2008 – 11/2013	None
Pracon et al	05/2014 – 11/2019	None
Abramovitz Fuoks et al	01/2015 – 10/2021	None
Maarse et al	2010 – 2022	None
Galea et al	06/2015 – 12/2023	None
Fukushima et al	09/2021 – 01/2023	Supported by Edwards Lifesciences, Medtronic, Boston Scientific, Abbott Medical and Daiichi-Sankyo

Table S3. Control group population

**Source**	
Masjuan et al	/
Galloo et al	/
Cruz-Gonzalez et al	LAAC patients with other indications than stroke on OAC
Pracon et al	LAAC patients with bleeding as primary indication
Abramovitz Fuoks et al	/
Maarse et al	PSM of patients with breakthrough stroke continued with OAC
Galea et al	/
Fukushima et al	LAAC patients with no history of IS

IS: Ischemic Stroke; LAAC: Left Atrial Appendage Closure; OAC: Oral Anticoagulation; PSM: Propensity-Score Matching

Table S4. LAAC-Device used

Source	Watchman 2.5, n (%)	Watchman FLX, n (%)	ACP/Amulet, n (%)	Others, n (%)
Masjuan et al	0 (0)	0 (0)	19 (100)	0 (0)
Galloo et al	4 (27)	0 (0)	11 (73)	0 (0)
Cruz-Gonzalez et al	0 (0)	0 (0)	115 (100)	0 (0)
Pracon et al	17 (43.6)	0 (0)	22 (56.4)	0 (0)
Abramovitz Fuoks et al	16 (55.2)	13 (44.8)	0 (0)	0 (0)
Maarse et al	135 (31)	97 (22)	187 (43)	17 (4)
Galea et al	10 (11)	31 (33)	40 (42)	14 (14)
Fukushima et al	106 (30.6)	240 (69.4)	0 (0)	0 (0)

Table S5. Percentage of patients on direct oral anticoagulation (DOAC) or vitamin K antagonist (VKA) at discharge and at follow-up

	**Discharge**		**Follow-up**	
**Source**	**DOAC, n (%)**	**VKA, n (%)**	**DOAC, n (%)**	**VKA, n (%)**
Masjuan et al	17 (89.5)	2 (10.5)	17 (89.5)	2 (10.5)
Galloo et al	7 (46.6)	4 (26.7)	NA	NA
Cruz-Gonzalez et al	2 (1.7)	46 (40)	3 (2.7)	6 (5.4)
Pracon et al	0 (0)	0 (0)	NA	NA
Abramovitz Fuoks et al	NA	NA	NA	NA
Maarse et al	132 (30.2)	107 (24.5)	NA	NA
Galea et al	NA	NA	NA	NA
Fukushima et al	NA	NA	NA	NA

Table S6. Clinical outcomes at mean/median follow-up

Source	All-cause death, n (%)	Cardiovascular death, n (%)	Hemorrhagic stroke, n (%)	DRT, n (%)
Masjuan et al	0 (0)	0 (0)	NA	0 (0)
Galloo et al	3 (20)	0 (0)	0 (0)	0 (0)
Cruz-Gonzalez et al	8 (7.2)	NA	NA	4 (5.2)
Pracon et al	3 (7.7)	NA	NA	NA (13.20)
Abramovitz Fuoks et al	1 (3.4)	NA	NA	0 (0)
Maarse et al	25 (5.8)	NA	NA	24 (6)
Galea et al	5 (5)	NA	NA	2 (2)
Fukushima et al	22 (6.4)	12 (3.5)	1 (0.3)	10 (2.9)

Table S7. Periprocedural safety

**Source**	**Periprocedural safety, n (%)**	**Remarks**
Masjuan et al	0 (0)	
Galloo et al	1 (6.7)	One episode of device embolization
Cruz-Gonzalez et al	NA (7.8)	
Pracon et al	NA	Procedure related clinically significant bleeding (n=2; 5.2%)
Abramovitz Fuoks et al	0 (0)	
Maarse et al	31 (7.0)	
Galea et al	1 (1)	One episode of TIA
Fukushima et al	5 (1.5)	

PROTOCOL AMENDMENT

Date of Amendment	Section of Change	Original	Revision	Rationale
12.01.2026	Inclusion Criteria	Studies will be eligible for inclusion if they meet the following two criteria: (1) report the results of a study of original design, and (2) report the incidence of the outcome of stroke following LAAC in patients with **previous ischemic stroke** under oral anticoagulation. No restrictions will be considered in terms of publication year, language or sample size of the study. Case reports and case series will be excluded. When potentially overlapping study populations will be identified in different publications (according to the period of recruitment and involved institutions), we will give preference to the most recent publication or the report providing data on antithrombotic treatment at discharge and throughout the follow-up.	Studies will be eligible for inclusion if they meet the following two criteria: (1) report the results of a study of original design, and (2) report the incidence of the outcome of stroke following LAAC in patients with **previous thromboembolic event or LAA thrombus** under oral anticoagulation. No restrictions will be considered in terms of publication year, language or sample size of the study. Case reports and case series will be excluded. When potentially overlapping study populations will be identified in different publications (according to the period of recruitment and involved institutions), we will give preference to the most recent publication or the report providing data on antithrombotic treatment at discharge and throughout the follow-up.	The inclusion criteria were expanded to capture a broader and more representative patient population. Consequently, the search strategy was refined to align with the updated criteria and to ensure retrieval of key studies known a priori that were not identified using the initial search strategy.

PRIMSA 2020 FOR ABSTRACTS CHECKLIST

Section and Topic	Item #	Checklist item	Reported (Yes/No)
**TITLE**			
Title	1	Identify the report as a systematic review.	1-3
**BACKGROUND**			
Objectives	2	Provide an explicit statement of the main objective(s) or question(s) the review addresses.	46-48
**METHODS**			
Eligibility criteria	3	Specify the inclusion and exclusion criteria for the review.	49-50
Information sources	4	Specify the information sources (e.g. databases, registers) used to identify studies and the date when each was last searched.	51-52
Risk of bias	5	Specify the methods used to assess risk of bias in the included studies.	56-58
Synthesis of results	6	Specify the methods used to present and synthesise results.	54-56
**RESULTS**			
Included studies	7	Give the total number of included studies and participants and summarise relevant characteristics of studies.	59
Synthesis of results	8	Present results for main outcomes, preferably indicating the number of included studies and participants for each. If meta-analysis was done, report the summary estimate and confidence/credible interval. If comparing groups, indicate the direction of the effect (i.e. which group is favoured).	60-68
**DISCUSSION**			
Limitations of evidence	9	Provide a brief summary of the limitations of the evidence included in the review (e.g. study risk of bias, inconsistency and imprecision).	71
Interpretation	10	Provide a general interpretation of the results and important implications.	69-73
**OTHER**			
Funding	11	Specify the primary source of funding for the review.	No
Registration	12	Provide the register name and registration number.	56

From: Page MJ, McKenzie JE, Bossuyt PM, Boutron I, Hoffmann TC, Mulrow CD, et al. The PRISMA 2020 statement: an updated guideline for reporting systematic reviews. BMJ 2021;372:n71. doi: 10.1136/bmj.n71. This work is licensed under CC BY 4.0. To view a copy of this license, visit https://creativecommons.org/licenses/by/4.0/

Table S8. PRISMA 2020 CHECKLIST

Section and Topic	Item #	Checklist item	Location where item is reported
**TITLE**			
Title	1	Identify the report as a systematic review.	1-3
**ABSTRACT**			
Abstract	2	See the PRISMA 2020 for Abstracts checklist.	done
**INTRODUCTION**			
Rationale	3	Describe the rationale for the review in the context of existing knowledge.	99-121
Objectives	4	Provide an explicit statement of the objective(s) or question(s) the review addresses.	122-124
**METHODS**			
Eligibility criteria	5	Specify the inclusion and exclusion criteria for the review and how studies were grouped for the syntheses.	144-151
Information sources	6	Specify all databases, registers, websites, organisations, reference lists and other sources searched or consulted to identify studies. Specify the date when each source was last searched or consulted.	133-139, Appendix
Search strategy	7	Present the full search strategies for all databases, registers and websites, including any filters and limits used.	Appendix
Selection process	8	Specify the methods used to decide whether a study met the inclusion criteria of the review, including how many reviewers screened each record and each report retrieved, whether they worked independently, and if applicable, details of automation tools used in the process.	139-141
Data collection process	9	Specify the methods used to collect data from reports, including how many reviewers collected data from each report, whether they worked independently, any processes for obtaining or confirming data from study investigators, and if applicable, details of automation tools used in the process.	154-156
Data items	10a	List and define all outcomes for which data were sought. Specify whether all results that were compatible with each outcome domain in each study were sought (e.g. for all measures, time points, analyses), and if not, the methods used to decide which results to collect.	156-168
	10b	List and define all other variables for which data were sought (e.g. participant and intervention characteristics, funding sources). Describe any assumptions made about any missing or unclear information.	NA
Study risk of bias assessment	11	Specify the methods used to assess risk of bias in the included studies, including details of the tool(s) used, how many reviewers assessed each study and whether they worked independently, and if applicable, details of automation tools used in the process.	171-173
Effect measures	12	Specify for each outcome the effect measure(s) (e.g. risk ratio, mean difference) used in the synthesis or presentation of results.	176-184
Synthesis methods	13a	Describe the processes used to decide which studies were eligible for each synthesis (e.g. tabulating the study intervention characteristics and comparing against the planned groups for each synthesis (item #5)).	144-151
	13b	Describe any methods required to prepare the data for presentation or synthesis, such as handling of missing summary statistics, or data conversions.	176-189
	13c	Describe any methods used to tabulate or visually display results of individual studies and syntheses.	184-185

Section and Topic	Item #	Checklist item	Location where item is reported
	13d	Describe any methods used to synthesize results and provide a rationale for the choice(s). If meta-analysis was performed, describe the model(s), method(s) to identify the presence and extent of statistical heterogeneity, and software package(s) used.	181-185
	13e	Describe any methods used to explore possible causes of heterogeneity among study results (e.g. subgroup analysis, meta-regression).	NA
	13f	Describe any sensitivity analyses conducted to assess robustness of the synthesized results.	NA
Reporting bias assessment	14	Describe any methods used to assess risk of bias due to missing results in a synthesis (arising from reporting biases).	NA
Certainty assessment	15	Describe any methods used to assess certainty (or confidence) in the body of evidence for an outcome.	NA
**RESULTS**			
Study selection	16a	Describe the results of the search and selection process, from the number of records identified in the search to the number of studies included in the review, ideally using a flow diagram.	192-195
	16b	Cite studies that might appear to meet the inclusion criteria, but which were excluded, and explain why they were excluded.	193-194
Study characteristics	17	Cite each included study and present its characteristics.	195-200, 208-232
Risk of bias in studies	18	Present assessments of risk of bias for each included study.	Appendix
Results of individual studies	19	For all outcomes, present, for each study: (a) summary statistics for each group (where appropriate) and (b) an effect estimate and its precision (e.g. confidence/credible interval), ideally using structured tables or plots.	468 (Fig. 2)
Results of syntheses	20a	For each synthesis, briefly summarise the characteristics and risk of bias among contributing studies.	201-207
	20b	Present results of all statistical syntheses conducted. If meta-analysis was done, present for each the summary estimate and its precision (e.g. confidence/credible interval) and measures of statistical heterogeneity. If comparing groups, describe the direction of the effect.	233-239
	20c	Present results of all investigations of possible causes of heterogeneity among study results.	237-238
	20d	Present results of all sensitivity analyses conducted to assess the robustness of the synthesized results.	NA
Reporting biases	21	Present assessments of risk of bias due to missing results (arising from reporting biases) for each synthesis assessed.	NA
Certainty of evidence	22	Present assessments of certainty (or confidence) in the body of evidence for each outcome assessed.	NA
**DISCUSSION**			
Discussion	23a	Provide a general interpretation of the results in the context of other evidence.	244-287
	23b	Discuss any limitations of the evidence included in the review.	307-320
	23c	Discuss any limitations of the review processes used.	307-320
	23d	Discuss implications of the results for practice, policy, and future research.	288-304

Section and Topic	Item #	Checklist item	Location where item is reported
**OTHER INFORMATION**			
Registration and protocol	24a	Provide registration information for the review, including register name and registration number, or state that the review was not registered.	128
	24b	Indicate where the review protocol can be accessed, or state that a protocol was not prepared.	128
	24c	Describe and explain any amendments to information provided at registration or in the protocol.	Appendix
Support	25	Describe sources of financial or non-financial support for the review, and the role of the funders or sponsors in the review.	40
Competing interests	26	Declare any competing interests of review authors.	29-39
Availability of data, code and other materials	27	Report which of the following are publicly available and where they can be found: template data collection forms; data extracted from included studies; data used for all analyses; analytic code; any other materials used in the review.	NA

From: Page MJ, McKenzie JE, Bossuyt PM, Boutron I, Hoffmann TC, Mulrow CD, et al. The PRISMA 2020 statement: an updated guideline for reporting systematic reviews. BMJ 2021;372:n71. doi: 10.1136/bmj.n71. This work is licensed under CC BY 4.0. To view a copy of this license, visit https://creativecommons.org/licenses/by/4.0/
